# Supplementary material for: Deep learning-based optical coherence tomography angiography image construction using spatial vascular connectivity network
Source: Commun Eng. 2024 Feb 9;3:28. doi: 10.1038/s44172-024-00173-9 (PMC10955818; doi:10.1038/s44172-024-00173-9)
Supplement: Supplementary file 2 — Supplementary Information [file 44172_2024_173_MOESM2_ESM.pdf]

Supplementary Information for

Deep learning-based optical coherence tomography angiography image construction using spatial vascular connectivity network

David Le, Taeyoon Son, Tae-Hoon Kim, Tobiloba Adejumo, Mansour Abtahi, Shaiban Ahmed, Alfa Rossi, Behrouz Ebrahimi, Albert Dadzie, Guangying Ma, Jennifer I. Lim, and Xincheng Yao

Supplementary Notes 1: Animal OCT Acquisition

For animal data acquisition, this study collected OCT and OCTA data from mice eyes. Briefly, the mouse custom SD OCT system utilized a near-infrared (NIR) superluminescent diode (SLD; λ = 810 nm; Δλ= 100 nm; Superlum, Carrigtwohill, County Cork, Ireland) light source. A line CCD camera with 2048 pixels (AViiVA EM4; e2v Technologies, Chelmsford, United Kingdom) was used for recording OCT spectra in the custom-build OCT spectrometer. The frame rate of the camera was set to 50 kHz. The axial and lateral resolution were theoretically estimated as 2.9 and 11 µm, respectively. The power illuminated at the mouse cornea was ~1 mW.

For the image acquisition of wild type mice (strain: C57BL/6J), an anesthetic agent was intraperitoneally induced by a mixture of ketamine (100 mg / kg body weight) and xylazine (5 mg / kg body weight) and a drop of 1% tropicamide ophthalmic solution (Akorn, Lake Forest, Illinois) was applied to the imaging eye. Next, a cover glass (12-545-80; Microscope over glass, Fisherbrand, Waltham, Massachusetts) with a drop of eye gel (Severe; GenTeal, Novartis, Basel, Switzerland) was placed on the imaging eye. After the mouse was completely anesthetized, the head was fixed by a bite bar and ear bar in the animal holder that provided five degrees of freedom (i.e., x, y, z, pitch, and roll). Volumetric raster scans were acquired from the various retinal quadrants, e.g., dorsal, ventral. Four repeated B-scans at each slow-scan position were collected for OCTA construction; thus, each OCT volume consisted of 4 × 600 × 600 A-scans and covered a FOV of 1.2 × 1.2 mm^2.

For the animal dataset, we collected a total of 24 OCT datasets from 6 mice, corresponding to one eye and four retinal quadrants, i.e., dorsal, nasal, ventral, and temporal. Datasets that had inhomogeneous brightness and other imaging artifacts were excluded. Therefore, 16 volumes comprised the dataset, 9 volumes for training, 1 volume for validation, and 6 volumes for testing. Equivalently, since each volume contained 598 B-scans, namely due to the exclusion of the first and last B-scans as they cannot be used to generate SVC inputs. The equivalent individual images for training, validation and testing were 5,382, 598 and 3,588 images, respectively.

Supplementary Notes 2: Human OCT Acquisition

Briefly, the human custom SD OCT system utilized the same NIR SLD λ = 810 nm; Δλ= 100 nm; Superlum, Carrigtwohill, County Cork, Ireland). A line CCD camera with 2048 pixels (AViiVA EM4; e2v Technologies, Chelmsford, United Kingdom) was used for recording OCT spectra in the custom-build OCT. spectrometer. The frame rate of the camera was set to 70 kHz. The axial and lateral resolution were theoretically estimated at 1 and 10 µm, respectively. The illumination power on the human cornea was ~600 µW. For the image acquisition of human subjects, no anesthetic agent was used. A custom chin rest was employed to reduce head movements, a fixation target with a dim red light was used to minimize voluntary eye movements, and a pupil camera was used to aid in retinal localization by the photographer. Volumetric

raster scans were acquired from the macular. Four repeated B-scans at each slow-scan position were recollected for OCTA construction; thus, each OCT volume consisted of 4 × 300 × 300 A-scans and covered a FOV of 3 × 3 mm^2.

For this dataset, we collected a total of 16 eyes from 8 healthy subjects. Datasets with inhomogeneous brightness and severe motion artifact were excluded, for this study 10 eyes comprised the dataset, since each eye contains 4 OCT volumes, all of the repeated volumes were used for training as a form of data augmentation for the training dataset. Therefore, 16 volumes were used for training, 1 volume for validation and 6 volumes for testing. Equivalently, since each volume contained 298 B-scans, namely due to the exclusion of the first and last B-scans as they cannot be used to generate SVC inputs. The equivalent individual images for training, validation and testing were 4,768, 298 and 1490 images, respectively.

Additionally, three eyes from three patients with proliferative diabetic retinopathy (PDR) was imaged. Images that contained poor signal quality were excluded. Only one retinopathy eye was used to evaluate our model. The patient was recruited from the UIC Retinal Clinic. All recruited patients underwent complete anterior and dilated posterior segment examination by an experienced ophthalmologist (JIL). They also underwent contact lens examination with a slit lamp to identify PDR signs. The patients were classified based on the severity of DR (mild, moderate and severe NPDR, and PDR) according to the Early Treatment Diabetic Retinopathy Study (ETDRS) staging system. According to the American Academy of Ophthalmology, the ETDRS levels are classified as the following: no apparent retinopathy (Level 10), mild NPDR (Level 20), moderate NPDR (Level 35), severe NPDR (Level 53), and PDR (level 61) [42].The patient was imaged using the custom OCT system and protocol.

Supplementary Notes 3: SV Processing

The OCT scan pre-processing starts registration of the OCT volume. The method that was employed for frame registration the Discrete Fourier Transform (DFT) registration method [37]. Since the OCT volume contains multiple repeated scans, the first step is to perform intra-frame registration, where each repetitive scan is registered to the first scan. This process is repeated for all scans. Next, inter-frame registration is performed to register each of the scans within the volume. After the OCT Volume Pre-processing, OCTA images were constructed by implementing an intensity-based speckle variance (SV) processing for both mouse and human OCT volumes with the following equation [38]:

$$SV_{ij} = \frac{1}{N}\sum_{i}^{N}\left[I_{ijk}(x,z) - \frac{1}{N}\sum_{i}^{N} I_{ijk}(x,z)\right]^2 = \frac{1}{N}\sum_{i}^{N}\left[I_{ijk} - (I_{mean})_{jk}\right]^2 \quad (13)$$

Where i, j and k are indices of frame, lateral and depth pixel of the OCT B-scan, respectively. N is the number of frames used in the calculation. $(I_{mean})_{jk}$ is the averaged frame of N frames over the sample pixel.

Supplementary Notes 4: Model Training

The training procedure utilized the Adam optimizer with a learning rate of 0.0001, the loss function used to train the model was either the MSE or SSIM, and a batch size of 32. Transfer learning was employed using pretrained weights from the ImageNet Dataset for the encoder, and the decoder was initialized with random weights. To promote stable training between different models and datasets, data augmentation, in the form of, i.e., horizontal flips, zoom, vertical and horizontal shifting, was implemented. The deep learning implementation was on a workstation with Ubuntu operating system (v20.04). Three NVIDIA

Quadro 6000, graphics processing units (GPU) were utilized. All deep learning implementations were performed using Keras API with Tensorflow backend (v2.9.1).

Supplementary Notes 5: Loss Function

Mean-Squared Error Loss

The formulation of MSE is detailed as follows:

$$L_{MSE} = MSE = \frac{1}{MN}\sum_{i=1}^{M}\sum_{j=1}^{N}\left(X_{ij} - Y_{ij}\right)^2 \quad (1)$$

Where $(X_{ij} - Y_{ij})$ denote the pixel-wise error difference between the predicted image, X, and the ground truth, Y, and M, N denotes the number of rows and columns in the images [39].

Structural Similarity Loss

The SSIM is a perceptually motivated function that takes into comparison distinct aspects of the image that is inspired by the human visual system (HVS), that is the luminance, contrast, and structure.

For two image patches, x and y, being compared, the luminance parameter can be determined by:

$$l(x,y) = \frac{2\mu_x\mu_y + C_1}{\mu_x^2 + \mu_y^2 + C_1} \quad (2)$$

The contrast parameter can be determined by:

$$c(x,y) = \frac{2\sigma_x\sigma_y + C_2}{\sigma_x^2 + \sigma_y^2 + C_2} \quad (3)$$

The structural parameter is defined by:

$$s(x,y) = \frac{\sigma_{xy} + C_3}{\sigma_x\sigma_y + C_3} \quad (4)$$

Where, μ_x and μ_y represents the mean, σ_x and σ_y represents the variance, and σ_{xy} represents the covariance. The C_1, C_2, and C_3 are small regularization constants to avoid instability for image regions with local mean or standard deviation close to zero and are determined as follows:

$$C_1 = (0.01 * L)^2 \quad (5)$$

$$C_2 = (0.03 * L)^2 \quad (6)$$

$$C_3 = \frac{C_2}{2}, \quad (7)$$

Where L is the dynamic range of the image, e.g., for data type uint8, $L = 255$.

Therefore, combining the different parameters together, the SSIM can be defined as:

$$SSIM(x,y) = [l(x,y)]^{\alpha} \cdot [c(x,y)]^{\beta} \cdot [s(x,y)]^{\gamma} \quad (8)$$

Where x and y are the predicted and ground truth images, respectively. Variables, α, β, and γ are parameters to define the relative importance of the three components. For the purpose of this study, $\alpha = \beta = \gamma = 1$. SSIM can also be written as the following:

$$SSIM(x, y) = \frac{(2\mu_x\mu_y + C_1)(2\sigma_{xy} + C_2)}{(\mu_x^2 + \mu_y^2 + C_1)(\sigma_x^2 + \sigma_y^2 + C_2)} \quad (9)$$

To formulate SSIM as a loss function, we define it as follows:

$$L_{SSIM} = 1 - SSIM(x, y) \quad (10)$$

Supplementary Notes 6: Evaluation metrics

The Peak Signal to Noise Ratio (PSNR) is defined as a relationship between the maximum intensity value and the mean-squared-error. Therefore, the PSNR can be determined as:

$$PSNR = 10 \cdot \log\left(\frac{Max_I^2}{MSE}\right) \quad (11)$$

Where the Max_I is the maximum pixel intensity, i.e., for a uint8 image is 255, and MSE is formulated in (1).

The Multiscale Structural Similarity Index Measure (MS-SIM) is an extension of SSIM, which utilizes three different visual perception parameters, namely the luminance, contrast, and structural parameters for localized regions. The MS-SSIM evaluates SSIM at multiple scales, i.e., at different resolutions and visual perceptions. In order to obtain MS-SSIM, an iterative approach is employed where the reference and the output images are scaled M-1 times and down sampled by a factor of two after each iteration. The contrast and structural parameter are calculated at each scale while the luminance parameter is computed only at the M-th scale. The formulation of MS-SSIM is as follows:

$$MS - SSIM(x, y) = [l_M(x, y)]^{\alpha_M} \cdot \prod_{j=1}^{M} [c_j(x, y)]^{\beta_j} [s_j(x, y)]^{\gamma_j} \quad (12)$$

Supplementary Notes 7: En face Projection

For en face images, the superficial vascular plexus (SVP) is demarcated by the NFL-GCL and the IPL-INL boundaries, and the deep vascular plexus (DVP) is demarcated by the IPL-INL and the OPL-ONL boundaries. For mouse dataset, retinal flattening was performed by realigning each A-line. Since the retinal quadrants, e.g., dorsal, of the mouse retina was smooth, the boundaries of the SVP and DVP were manually segmented. For human dataset, retinal layer segmentation was performed using the Iowa Reference Algorithms (Retinal Image Analysis Lab, Iowa Institute for Biomedical Imaging, Iowa City, IA) to determine the boundaries for the SVP and DVP [43-45]. The en face images were generated using average intensity projection. Image processing was performed on MATLAB R2021b (MathWorks, Natick, Massachusetts) with image processing packages in ImageJ [46].
